# RNA binding proteins regulate anabolic and catabolic gene expression in chondrocytes

**DOI:** 10.1016/j.joca.2016.01.988

**Published:** 2016-07

**Authors:** B.T. McDermott, S. Ellis, G. Bou-Gharios, P.D. Clegg, S.R. Tew

**Affiliations:** Department of Musculoskeletal Biology, Institute of Ageing and Chronic Disease, University of Liverpool, Leahurst Campus, Neston, Cheshire CH64 7TE, UK

**Keywords:** Chondrocyte, mRNA, SOX9, MMP13

## Abstract

**Objective:**

Regulation of anabolic and catabolic factors is considered essential in maintaining the homoeostasis of healthy articular cartilage. In this study we investigated the influence of RNA binding proteins (RNABPs) in this process.

**Design:**

Using small interfering RNA (siRNA), RNABP expression was knocked down in SW1353 chondrosarcoma cells and human articular chondrocytes. Gene expression and messenger RNA (mRNA) decay of anabolic (SOX9, Aggrecan) and catabolic (matrix metalloproteinase (MMP)13) factors were analysed using reverse transcription quantitative polymerase chain reaction (RT-qPCR). RNA-electromobility shift assays (EMSAs) were used to investigate RNABP interactions with the SOX9 mRNA 3′ untranslated region (UTR). Immunohistochemical localisation of MMP13 and the RNABP human antigen R (HuR) was performed in E13.5 and E16.5 mouse embryo sections.

**Results:**

SOX9 mRNA, mRNA half-life and protein expression were increased with siRNA targeting the RNABP tristetraprolin (TTP) in both HACs and SW1353s. TTP knockdown also stimulated aggrecan mRNA expression but did not affect its stability. RNA-EMSAs demonstrated that adenine uracil (AU)-rich elements in the SOX9 mRNA 3′UTR interacted with chondrocyte proteins with three specific elements interacting with TTP. HuR knockdown significantly increased MMP13 expression and also regulated the expression of a number of known transcriptional repressors of MMP13. HuR was ubiquitously expressed within mouse embryos yet displayed regional down-regulation within developing skeletal structures.

**Conclusion:**

This study demonstrates for the first time how RNABPs are able to affect the balance of anabolic and catabolic gene expression in human chondrocytes. The post-transcriptional mechanisms controlled by RNABPs present novel avenues of regulation and potential points of intervention for controlling the expression of SOX9 and MMP13 in chondrocytes.

## Introduction

Articular chondrocytes are responsible for the maintenance of the highly specialised extracellular matrix (ECM) of articular cartilage, found within diarthrodial joints and their phenotype is characterised by the expression of cartilage ECM components such as collagen type II and aggrecan^1^. Both of these genes are regulated by SOX9, a transcription factor, which is an essential component of transactivation complexes that encode for cartilage ECM components (for review see Ref. 2). Healthy cartilage tissue persists through the controlled breakdown and synthesis of ECM factors by chondrocytes. This breakdown is mediated by specialised proteases, such as those from the matrix metalloproteinase (MMP) family. One member of this family, the collagenase MMP13, has been particularly implicated in the breakdown of the cartilage collagen type II network^3^, and therefore articular surface destruction^4^.

The ability to regulate anabolic and catabolic factors in the joints has the scope to be of considerable importance in treating disorders of cartilaginous tissues. It is known that SOX9 is down regulated in osteoarthritic articular cartilage and ageing intervertebral disc^5^, ^6^. Forced expression of SOX9 in chondrocytes^7^ or in cells from the nucleus pulposus^8^ promotes the formation of cartilaginous ECM demonstrating that driving SOX9 expression forms an important component of strategies to regenerate cartilaginous tissues. Conversely, inhibition of MMP13 can prevent cartilage degradation without causing musculoskeletal syndrome joint side effects seen previously in patients with non-selective MMP inhibitors^9^.

A number of studies have identified mechanisms of how anabolic and catabolic processes are regulated at the transcriptional level. For instance, genetic studies of SOX9 in humans and mice have revealed that the transcriptional regulation of SOX9 is very complex, involving both long range and proximal regulatory elements^10^, ^11^, ^12^, ^13^, ^14^. The proximal promoter of MMP13 has also been well characterised, identifying binding of both transactivators and transcriptional repressors^15^, ^16^. In addition to transcriptional control, post-translational control of both anabolic and catabolic factors in cartilage is important for controlling their function. For instance, the SOX9 protein can be phosphorylated or sumoylated, resulting in altered transactivation ability and proteasomal degradation respectively^17^, ^18^. In addition, it has been well established that MMP activity can be controlled through conversion of the enzymes from inactive pro-forms and by interactions with inhibitors such as the tissue inhibitors of metalloproteinases (TIMPs)^19^, ^20^.

A further potential tier of regulation is post-transcriptional regulation of messenger RNAs (mRNAs), a complex process controlled interactions of microRNAs (miRNAs) and RNA binding proteins (RNABPs) with target mRNAs. These interactions can alter mRNA half-life and are often closely linked to translation control as well^21^. miRNA and RNABP binding often occur at sites within the 3′ untranslated region (UTR) of the target mRNAs. These regions contain seeding sites for miRNAs and many also contain adenine uracil (AU)-rich elements (AREs), which often harbour binding sites for RNABPs such as tristetraprolin (TTP), human antigen R (HuR), KH RNA binding protein (KSRP) and AU-rich element ribonucleic acid (RNA) binding protein 1 (AUF-1)^22^.

We have been interested in the control of chondrocyte genes post-transcriptionally and have demonstrated that a number of mRNAs exhibit altered decay rates in osteoarthritic chondrocytes compared to those from healthy tissue. Furthermore we have separately shown that SOX9 expression can be controlled post-transcriptionally in response to cellular stress and that its mRNA half-life negatively correlates with overall SOX9 mRNA levels in chondrogenic constructs derived from both human articular chondrocyte (HAC) and human bone marrow derived stem cells^23^, ^24^, ^25^. A role is emerging for miRNAs in the post-transcriptional control of chondrocyte genes such as SOX9^26^, ^27^ but a clear role for RNABP regulation of gene expression in these cells is not yet established. Interestingly however, HuR knockout during murine embryonic development leads to a severe skeletal dysplasia^28^. This study therefore aimed to determine the role that RNABPs may play in the regulation of anabolic and catabolic mRNA levels in chondrocytes *in vitro*.

## Materials and methods

### Cell culture

Osteoarthritic human articular cartilage was obtained following total knee arthroplasty with full approval from the Cheshire Research Ethics Committee. Primary articular chondrocytes were isolated from tissue, dissected from intact, non-fibrillated areas of the articular surface by overnight digestion in growth media (Dulbecco's Modified Eagles Medium (DMEM) containing 10% Foetal Bovine Serum (FBS), 100 units/ml penicillin, 100 units/ml streptomycin, 5 μg/ml amphotericin B) supplemented with 0.08% collagenase type II (all from Invitrogen, Paisley, UK). Cells were plated at high density (1 × 10^5^ cells/cm^2^) and cultured in growth media before being used at the end of first or second passage. SW1353 chondrosarcoma cells were maintained in the same growth media before their use in the experiments. All cell culture was carried out at 37°C in a 5% CO_2_ environment.

### Small interfering RNA (siRNA) mediated gene knockdown

SW1353 cells or HACs were grown to 90–95% confluence and transfected with 10 pmol/cm^2^ control siRNA (product sc-37007, Santa Cruz Biotechnology, Santa Cruz, CA) or siRNA targeting TTP (Life Technologies, Warrington, UK), KSRP, HuR or AUF-1 (Santa Cruz Biotechnology, Santa Cruz, CA) to cause RNABP knockdown. For multiple knockdown of RNABP, overall siRNA exposure in all conditions was normalised to that of a triple knockdown by appropriate addition of control siRNA to a final overall siRNA concentration of 30 pmol/cm^2^. All transfections were carried out using Lipofectamine 2000 (Invitrogen, Paisley, UK) following manufacturer's instructions with the cells cultured in growth medium with penicillin, streptomycin and amphotericin B omitted. Cultures were analysed 24–48-h after transfection.

### Real time polymerase chain reaction (PCR)

Total RNA was isolated from cell cultures using Tri Reagent (Sigma, Poole, UK) and reverse transcribed using Molony-murine leukemia virus (M-MLV) reverse transcriptase, primed using random primers (Promega, Madison, WI). Real time PCR was performed on an ABI 7300 system using GoTaq^®^ qPCR Master Mix (Promega). Expression levels were calculated by the 2^−^^Δ^^Ct^ method^29^ with glyceraldehyde 3-phosphate dehydrogenase (GAPDH) as the reference expression gene using primers described in Table I.

### Western blotting

Chondrocyte cell lysates were extracted using 1× reducing sodium dodecyl sulfate (SDS) sample buffer, separated on 4–12% NuPAGE gels (Invitrogen) and blotted onto nitrocellulose. Blots were probed with antibodies recognising SOX9 (AB5535 from Merck-Millipore, Watford, UK), TTP (Sak21 a further gift from Dr Andrew Clark), HuR (antibody 19F12, Santa Cruz Biotechnology, Santa Cruz, CA), KSRP (antibody Ab33291, Abcam, Cambridge, UK), AUF-1 (a kind gift from Dr Gary Brewer, University of Medicine and Dentistry of New Jersey, USA) and GAPDH (Sigma, Poole, UK) using previously described protocols^24^. Blots were visualised using a UVP ChemiDoc-It Imaging System.

### RNA decay analysis

mRNA decay rate was determined in SW1353 cells 48 h after siRNA transfection. Real time PCR was used to quantify expression levels in complementary DNA (cDNA) generated from cells cultured with 1 μM actinomycin D (Sigma) for different times^24^.

### Generation of biotinylated RNA probes

Anti-sense deoxyribonucleic acid (DNA) templates, complimentary to the probe sequences in Table II, were purchased from Eurogentec. Templates encoding mutated versions of probes 5 and 6 were also obtained where the AUUUA region of the final RNA would be changed to AGGGA. The 3′ end of each DNA template included the following anti-sense T7 sequence – CCCTATAGTGAGTCGTATTA. A sense T7 oligo (TAATACGACTCACTATAGGG) was annealed to each of the SOX9 3′UTR templates at equimolar concentrations in annealing buffer (10 mM Tris, 50 mM NaCl, 1 mM Ethylenediaminetetraacetic acid (EDTA)) by heating at 95°C for 5 min and allowing the probes to cool slowly to room temperature before storing at 4°C. A T7 High Yield RNA Synthesis Kit (New England Biolabs, Hertfordshire, UK) was then used for *in vitro* transcription of the RNA probes according to the manufacturer's instructions. RNA was then purified using phenol chloroform and ethanol precipitation. To create probes for electromobility shift assay (EMSA), the RNAs were 3′ biotinylated with T4 RNA ligase using a RNA 3′ End Biotinylation Kit (Thermo Scientific, MA, USA) according to the manufacturer's instructions.

### RNA-EMSAs

Confluent HAC monolayer cultures were washed twice with phosphate buffered saline (PBS) and lysates prepared in EMSA extraction buffer (20 mM 4-(2-hydroxyethyl)-1-piperazineethanesulfonic acid (HEPES), 150 mM KCl, 4 mM MgCl_2_, 0.5 mM dithiothreitol (DTT), 0.5% Nonidet P-40 (Sigma) and complete protease inhibitor cocktail tablet (Roche, Basel, Switzerland)) using a cell scraper. The lysates were incubated with 40 ng of the biotinylated RNA probes in binding buffer (10 mM Tris HCl pH 7.5, 50 mM KCl, 1 mM DTT), 5% glycerol and 500 μg/ml transfer RNA (tRNA) in a final volume of 20 μl. The reaction mixture was incubated at 4°C for 20 min before the addition of 5 μl 5× loading buffer (50 mM Tris HCl pH 6.8, 0.01% bromophenol blue, 25% glycerol). Samples were then resolved by electrophoresis on a 0.5% Tris–borate–EDTA non-denaturing 6% polyacrylamide gel (Life Technologies, CA, USA) at 200 V for 1 h on ice. Probes and proteins in the gel were then transferred to Biodyne B nylon membranes (0.45 μM, Thermo Scientific) at 400 mA for 30 min on ice. Ultraviolet (UV) crosslinking was then performed on the membranes at 0.120 J/cm^2^. Biotinylated RNA probes were detected using a chemiluminescent nucleic acid detection kit (Thermo Scientific) according to the manufacturer's instructions and signal detection was achieved by exposing the membranes to a charge-coupled device (CCD) camera.

For recombinant TTP EMSAs, biotinylated RNA probes were either incubated with recombinant GST-TTP (Abnova) or with glutathione S-transferase (GST) alone as control. For probe competitor EMSAs, HAC protein lysates were incubated for 20 min at 4°C with non-biotinylated RNA probes before the addition of the corresponding biotinylated RNA probes. Non-biotinylated competitor RNA probes were added at 0-, 1- and 10-fold excess.

### Immunohistochemistry

E13.5 or E16.5 CBAF1 mouse embryos were fixed in cold 4% paraformaldehyde for 30 min, processed into paraffin wax blocks and cut into 10 μm sections. The sections were stained with either anti HuR or anti MMP13 antibodies (N16 goat polyclonal and H230 rabbit polyclonal respectively from Santa Cruz Biotechnology, CA, USA) and localisation visualised using appropriate horseradish peroxidase-conjugated secondary antibodies with 3,3′-diaminobenzidine as a substrate. Sections were then dehydrated, cleared in xylene and permanently mounted. The sections were examined and photographed using a Nikon Eclipse 80i microscope.

### Statistical analysis

In siRNA experiments using SW1353 and HAC cells, expression levels were normalised to control conditions and data was analysed using either one-way analysis of variance (ANOVA) or repeated measures ANOVA followed by Dunnett's *post hoc* test, or one-sample *t* testing. Paired data sets were analysed using paired *t* test. All data was normally distributed as determined by studying Q–Q plots. Data analysis was performed using SPSS software (IBM Corporation).

## Results

### Altered TTP expression regulates SOX9 mRNA levels in human chondrocytes

**Fig. 1:**
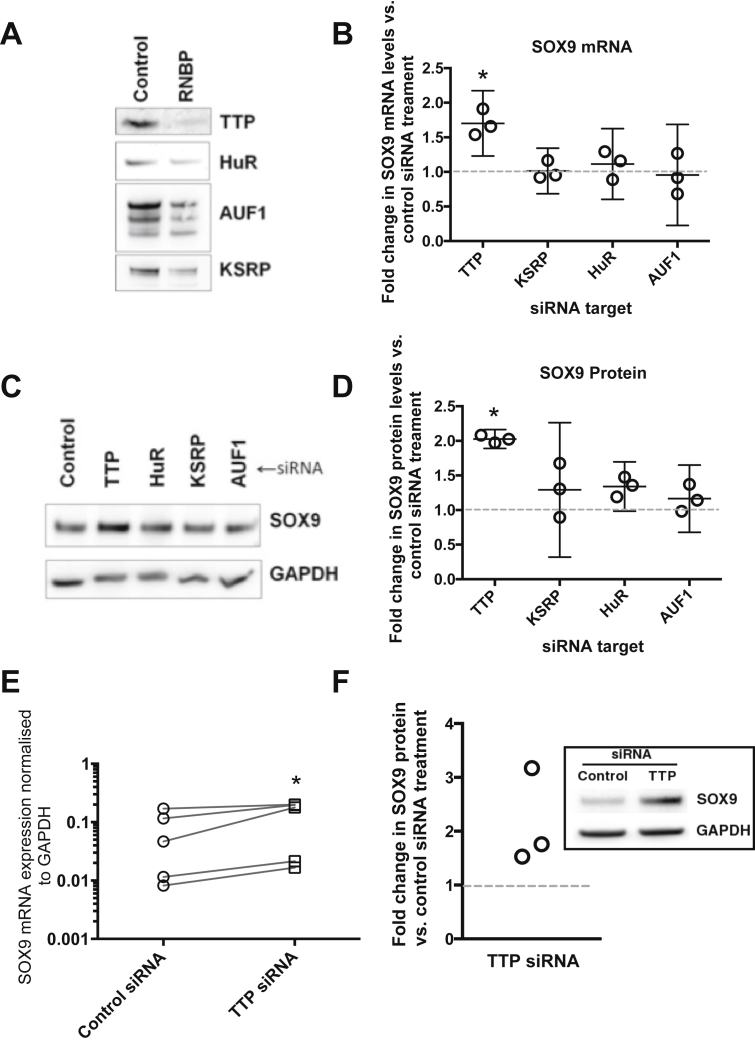
Knockdown of TTP leads to increased expression of SOX9 in human chondrocytic cells. (A) Western blot analysis of RNABP protein levels SW1353 cells treated with control siRNA or siRNAs targeting four specific RNABPs. (B) SOX9 mRNA expression levels in SW1353 cells treated with siRNAs targeting RNABPs. Data is normalised to GAPDH expression and to control siRNA expression (*n* = 3, individual data point presented with horizontal bar indicating mean and error bars indicating 95% confidence interval, *P = 0.006 one-way ANOVA, Dunnett's *post hoc* test). Grey dashed line in charts illustrates the normalised control level of mRNA expression. (C) Western blot analysis of SOX9 protein levels in SW1353 cells treated with siRNAs targeting RNABPs. (D) Normalised band intensity from western blots of SW1353 cells treated with siRNAs targeting RNABPs. Individual data points from three separate experiments are presented (horizontal bar indicates mean and error bars indicate 95% confidence intervals, *P = 0.025 one-way ANOVA, Dunnett's *post hoc* test). The grey dashed line illustrates the normalised control level of protein expression. (E) SOX9 mRNA expression levels in HAC treated with siRNA targeting TTP. Data is normalised to GAPDH expression. Individual data points from experiments from five separate donors presented with lines indicating paired samples (*P = 0.025 paired *t* test). (F) Western blot analysis of SOX9 protein levels in HACs treated with siRNA targeting TTP. A representative blot is shown (inset) as well as a chart illustrating normalised band intensity from blots generated from three separate experiments. The grey dashed line illustrates the normalised control level of protein expression.

To investigate whether RNABPs could be involved in chondrocyte mRNA regulation we knocked down their expression using a siRNA approach and examined expression of the mRNA and protein of the chondrocyte transcriptional regulator SOX9. In SW1353 chondrosarcoma cells, siRNA treatment resulted in knockdown of the targeted proteins to around 25% of control levels [Fig. 1(A)]. When SOX9 mRNA levels were examined in siRNA-treated SW1353 cells, we observed an increase in expression in cultures with reduced TTP expression [Fig. 1(B)]. We further examined SOX9 protein levels in these cultures and found that, consistent with the mRNA findings, SOX9 protein levels were increased when TTP expression was knocked down [Fig. 1(C)]. We extended our siRNA studies to examine HACs cultured as second passage monolayers and found in these cells that TTP knockdown increased the levels of both SOX9 mRNA [Fig. 1(D)] and SOX9 protein [Fig. 1(E)]. These results provide strong evidence that TTP can act as a negative regulator of SOX9 mRNA in chondrocytes.

### TTP knockdown alone is as effective as multiple RNABP knockdowns in regulating SOX9 mRNA expression

**Fig. 2:**
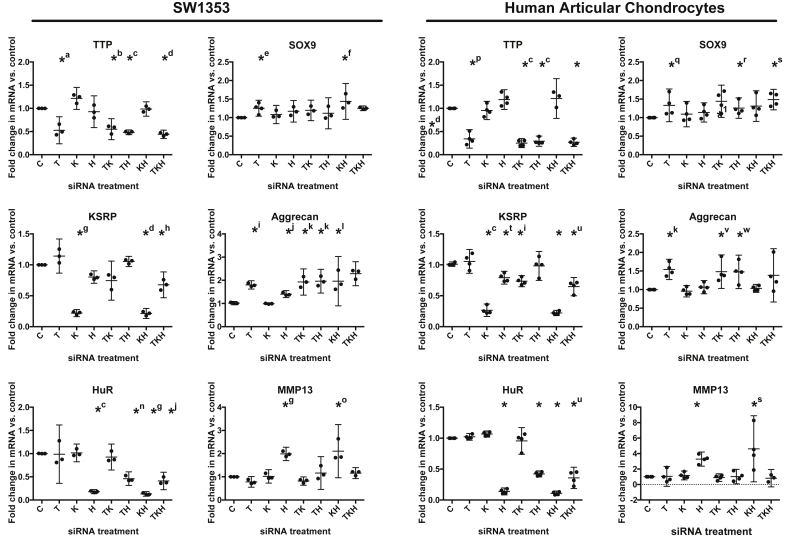
RNABP knockdown can regulate both anabolic and catabolic cartilage mRNAs. SW1353 (*n* = 3) HAC (*n* = 4) were transfected with TTP (T), HuR (H) and KSRP (K) siRNA either alone or in combinations with each other for 48 h before harvesting RNA for real time PCR analysis. TTP, KSRP, HuR, SOX9, aggrecan and MMP13 mRNA expression levels were normalised to GAPDH and then to control siRNA transfections. Individual data points presented as well as mean value (horizontal bar) and 95% confidence intervals (vertical error bars). *P < 0.001, *^a^*P* = 0.035, *^b^*P* = 0.027, *^c^*P* = 0.001, *^d^*P* = 0.003, *^e^*P* = 0.028, *^f^*P* = 0.044, *^g^*P* = 0.002, *^h^*P* = 0.032, *^i^*P* = 0.004, *^j^*P* = 0.013, *^k^*P* = 0.012, *^l^*P* = 0.041, *^m^*P* = 0.007, *^n^*P* = 0.009, *^o^*P* = 0.026, *^p^*P* = 0.008, *^q^*P* = 0.031, *^r^*P* = 0.045, *^s^*P* = 0.025, *^t^*P* = 0.010, *^u^*P* = 0.011, *^v^*P* = 0.043, *^w^*P* = 0.008, one-sample *t* test.

To examine the effect of RNABP knockdown on SOX9 expression in more detail, SW1353 and HACs were transfected with TTP siRNA either alone or in combination with HuR and KSRP siRNA. In both cell types, TTP, HuR and KSRP expression was significantly reduced when the RNABPs were knocked down alone and when knocked down in combination with each other (Fig. 2). Knockdown of HuR and KSRP was found to be less effective when TTP siRNA was co-transfected with either HuR or KSRP siRNA, suggesting TTP also regulates the expression of KSRP and HuR or influences the RNA interferance (RNAi) mechanism. TTP knockdown alone was shown to be as effective as multiple RNABP knockdowns in significantly increasing SOX9 mRNA expression in both SW1353 [Fig. 2(A)] and HACs [Fig. 2(B)].

### RNABP knockdown results in the regulation of both anabolic and catabolic chondrocyte mRNAs

In addition to the anabolic transcriptional regulator SOX9, we also examined the mRNA expression of the anabolic ECM component aggrecan and the catabolic collagenase MMP13 in chondrocytes where RNABP had been knocked down (Fig. 2). We found that aggrecan, like SOX9, was upregulated in cultures where TTP had been knocked down. Knockdown of multiple RNABPs did not attenuate the effect of TTP knockdown alone on aggrecan mRNA expression. MMP13 expression was not affected by TTP knockdown alone, but was instead significantly upregulated in cultures where HuR had been knocked down. However, induction of MMP13 was significantly and reproducibly attenuated, in cultures where TTP was knocked down alongside HuR (i.e., HuR + TTP or HuR + TTP + KSRP knockdown). This coincided with the impaired knockdown of HuR under these conditions. Responses to RNABP knockdown were comparable between SW1353 and HAC experiments on the whole although inconsistencies were evident. Notably these were in the responses of aggrecan where levels were elevated by all conditions except KSRP knockdown alone in SW1353 but only by knockdown of TTP alone, TTP + KSRP and TTP + HuR in HAC. A further inconsistency was the response of SOX9 to KSRP + HuR knockdown conditions where significant up-regulation was observed in SW1353 cells but not in HACs.

### TTP knockdown creates a more stable pool of SOX9 transcripts

**Fig. 3:**
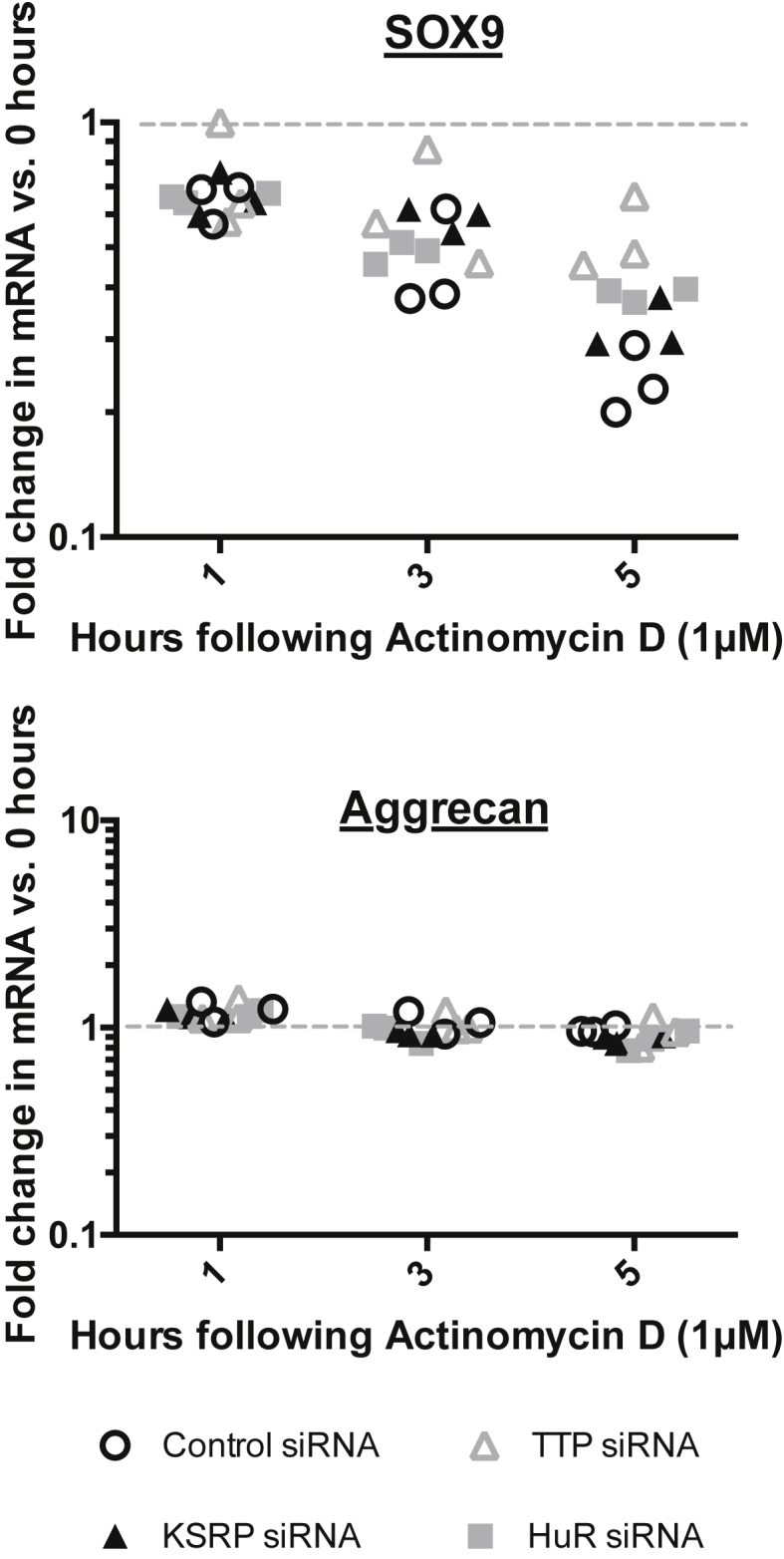
TTP knockdown stabilises SOX9 mRNA. SW1353 were transfected with Control (black unfilled circles), TTP (black unfilled triangles), KSRP (grey filled triangles) and HuR siRNA (grey unfilled squares) for 48 h and then subjected to a 5-h actinomycin D chase. RNA was harvested for real time PCR analysis for mRNA decay of SOX9 and aggrecan as indicated. Data is normalised to 0 h actinomycin D treatment (grey dashed line) and individual data points from three independent experiments are presented.

To determine whether TTP affects the post-transcriptional regulation of the anabolic mRNAs SOX9 and aggrecan, we performed actinomycin D chase experiments to determine the rate of decay of each mRNA in SW1353 cells following knockdown of the different RNABPs (Fig. 3). We found that SOX9 mRNA was turned over quickly, as we have previously observed^23^, ^24^. Interestingly, the rate of SOX9 mRNA decay appeared to be attenuated by TTP knockdown. By performing linear regression analysis of log2 transformed data on each replicate separately we found that SOX9 mRNA mean half-life under control conditions was 2.6 h (±1.3 95% confidence interval, *n* = 3) and that this was not significantly affected by knockdown of KSRP or HuR. However, TTP knockdown resulted in a stabilisation of the SOX9 mRNA, increasing its half-life to 6.2 h (±4.3 95% confidence interval, *n* = 3, *P* = 0.004 – ANOVA with Dunnett's *post hoc* test). Aggrecan mRNA was found to be very stable in the SW1353 cells (half-life > 10 h) and it was not detectably affected by knockdown of any of the RNABPs.

### Chondrocyte proteins interact with the SOX9 3′UTR

**Fig. 4:**
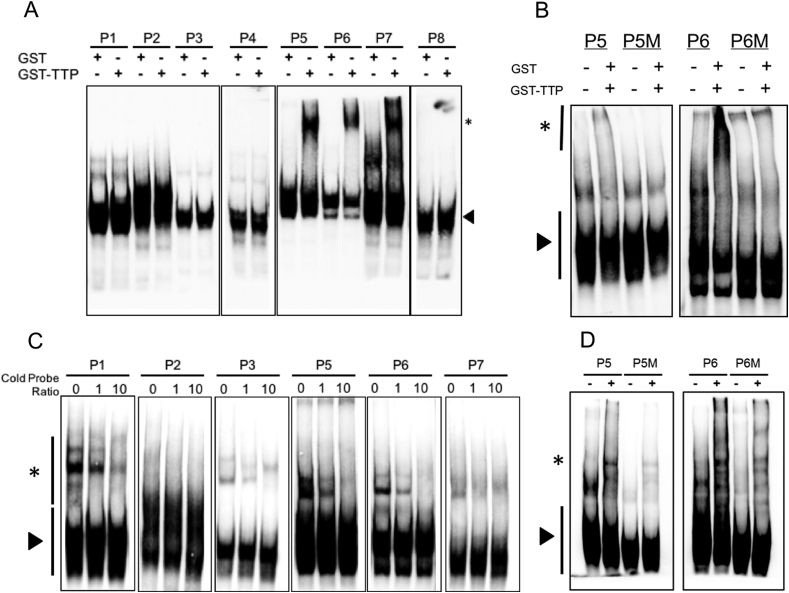
TTP interacts with SOX9 ARE-containing 3′UTR sequences. (A) EMSA was performed using the SOX9 3′UTR probes incubated with either GST or GST-TTP. Arrowhead indicates free probe and * indicates TTP-dependent gel shift. (B) EMSA with the SOX9 3′UTR probes 5 and 6 containing mutant AREs (P5M and P6M) incubated with either GST or GST-TTP. Arrowhead indicates free probe and * indicates TTP-dependent gel shift. (C) EMSA of six SOX9 3′UTR probes (numbers 1, 3, 5, 6 and 7) which demonstrated interactions with HAC protein lysates. Probes were incubated with 0-, 1- and 10-fold excess of non-biotinylated competitor probes prior to incubation with the lysate. Arrowhead indicates free probe and * indicates cell lysate induced gel shift. (D) EMSA of control (P5, P6) or mutant probes (P5M, P6M) incubated with (+) or without (−) HAC cell lysate. Arrowhead indicates free probe and * indicates cell lysate induced gel shift.

We were interested in studying how the 3′UTR of SOX9 can interact with proteins in chondrocytes. We have previously studied the sequence of this region of SOX9 mRNA and found that it contains AREs, specifically eight AUUUA pentamers^23^. We synthesised probes of approximately 100 bases in length spanning each motif (Table II) using *in vitro* transcription and then 3′ end labelled the probes with biotin. Three of the AREs (present in probes 5, 6 and 7) contain the sequence UAUUUAU and represent strong candidate TTP binding sites based on previous definitions^30^. To examine the role of TTP interactions with SOX9 3′UTR AREs, we performed EMSA analysis with probes incubated with recombinant TTP. We found that TTP interacted with probes 5, 6 and 7 only [Fig. 4(A)]. When the AUUUA pentamer was mutated in probe 5 and 6 to AGGGA, this interaction was lost [Fig. 4(B)]. Further EMSA analysis revealed that proteins within chondrocyte lysates interacted with five of the eight probes; probes 1, 3, 5, 6 and 7, and that this interaction could be inhibited in a dose-dependent manner with unlabelled competitor probes [Fig. 4(C)]. A sixth probe, probe 2, exhibited a faint and less convincing interaction with chondrocyte lysates. Using mutated probes 5 and 6 we found that whilst most of the cell lysate interactions were not dependent upon the AUUUA sequence, some high molecular weight complexes were diminished when using mutant probe 6 [Fig. 4(D)].

### HuR knockdown does not affect the decay rate of MMP13 but increases the expression of known transcriptional repressors

**Fig. 5:**
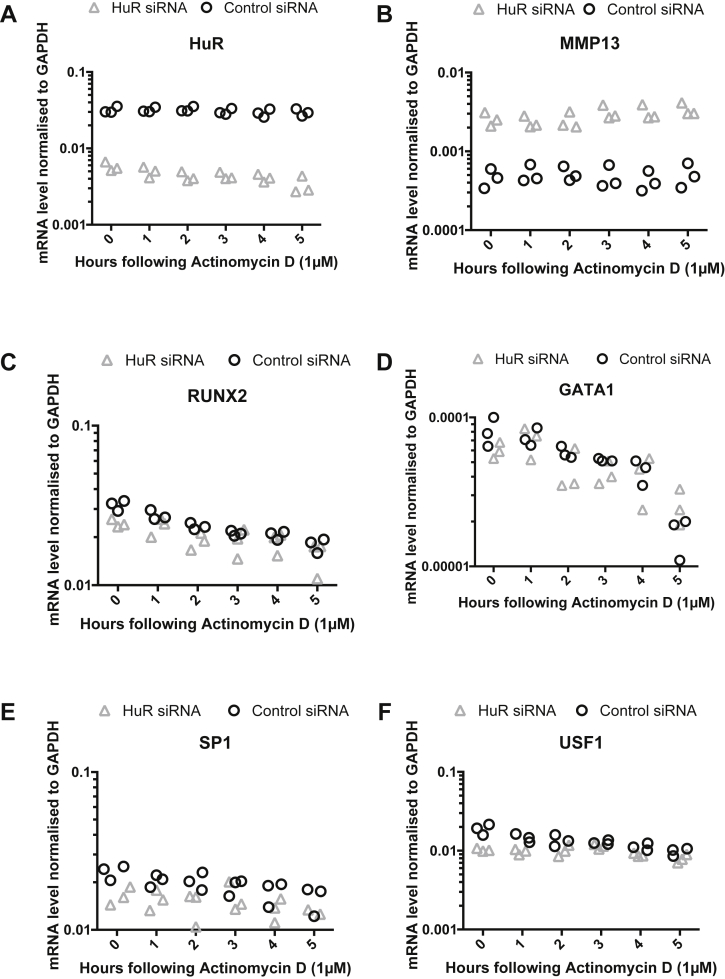
MMP13 mRNA stability and its transcriptional repressors are unaffected by HuR knockdown. SW1353 cells were transfected with either control or HuR siRNA and then subjected to a 6-h actinomycin D chase, prior to analysing mRNA stability using real time PCR. mRNA decay curves for (A) HuR and (B) MMP13 are presented in addition to those of the transcriptional regulators RUNX2 (C), GATA1 (D), Sp1 (E) and USF1 (F). Expression levels were normalised to GAPDH mRNA levels and are presented as individual data points on the charts (*n* = 3).

We examined the effect of RNABP knockdown on the mRNA decay of MMP13 in SW1353 and found that, like aggrecan, its transcript was very stable and that an affect of RNABP knockdown on turnover was not observed, despite overall mRNA levels being increased when HuR was knocked down [Fig. 5(B)]. Note that knockdown of HuR did not affect its own RNA stability [Fig. 5(A)]. Because MMP13 mRNA levels were increased when HuR was knocked down, we examined whether HuR had any effect on known transcriptional repressors of MMP13. We measured mRNA decay of four repressors in SW1353 cells [Fig. 5(B)]. We found an overall decrease in mRNA expression of RUNX2 and SP1 but no change in their mRNA decay rate in cells treated with HuR siRNA. A decrease in USF1 expression was also seen in the HuR knockdown cultures, which did seem to be associated with a stabilisation of this mRNA. GATA1 was not affected by HuR knockdown.

### HuR protein is spatially regulated in developing chondrocytes

**Fig. 6:**
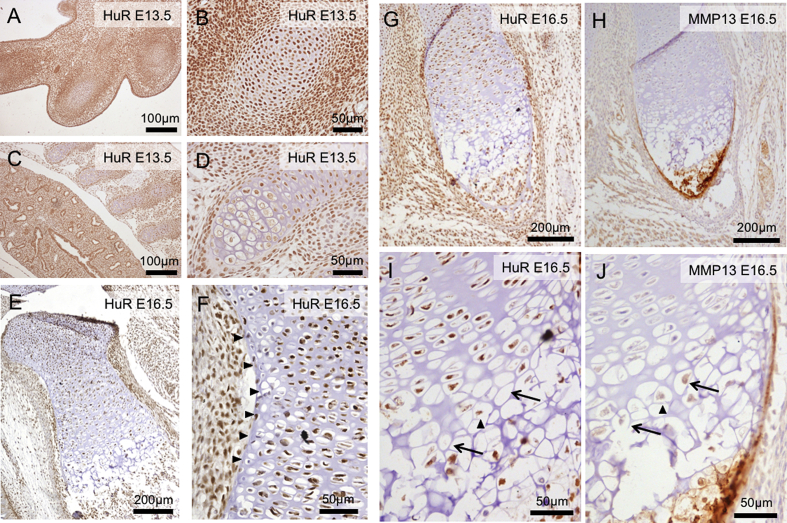
Immunohistochemical analysis of HuR and MMP13 distribution in embryonic murine cartilage. All antibody localisation was visualised using 3,3-diaminobenzidine (brown) and sections were counterstained with Haematoxylin (blue). (A) HuR localisation in the developing digits of the mouse forelimb at E13.5. A more highly magnified view of the central digit is shown in (B). (C) HuR localisation in the ribs and lung at E13.5. (D) HuR localisation in costal cartilage from E13.5 embryo. (E) HuR localisation in femur of E16.5 embryo. (F) Magnified view of E16.5 femur showing reduced staining at the margins of the cartilage rudiment (arrowheads). (G) HuR localisation in the radius of an E16.5 embryo. (H) Serial section of that shown in panel G, stained using MMP13 antiserum. (I & J) Magnified views of the hypertrophic chondrocyte regions shown in panels G and H respectively.

The regulation of MMP13 by HuR is particularly interesting given that MMP13 is expressed in terminally differentiating chondrocytes during endochondral ossification and that HuR knockout in mouse embryos causes a strong skeletal phenotype^28^. We decided to follow up on our observations by examining the protein localisation of HuR, and MMP13, in developing mouse cartilage at both E13.5 and E16.5 using immunohistochemistry. As has been well established, we found that, in general, HuR is expressed ubiquitously throughout the developing embryo. However we did note specific reductions in its abundance associated with cartilaginous structures. Firstly, we found that the condensing mesenchyme in the digits at E13.5 exhibited a “checkerboard” distribution of cells which were strongly HuR positive alongside cells with much weaker staining [Fig. 6(A and B)]. Furthermore, analysis of the more mature costal cartilage at E13.5, revealed that, compared to nearby lung tissue which mostly contained cells that highly express HuR, the rib cartilage can be separated into high expressing resting and proliferative cells and then much lower expressing hypertrophic cells [Fig. 6(B and D)]. We found similar evidence of reduced expression of HuR in hypertrophic chondrocytes in costal cartilage in E16.5 embryos (data not shown) and also in the long bones of the fore and hind limb [Fig. 6(E)]. Interestingly, we noted that HuR levels were low or absent in cells adjacent to the perichondrium in E16.5 long bones [Fig. 6(F)]. Using serial sections of E16.5 embryos we then examined the relationship between HuR and MMP13 protein expression. In the long bones we again observed that HuR expression was reduced in hypertrophic chondrocytes [Fig. 6(G and I)]. We found that MMP13 was located in the hypertrophic zone and that in earlier stages of differentiation was often in chondrocytes that had undetectable or reduced levels of HuR present [Fig. 6(H and I)]. The MMP13 in these reduced HuR cells was predominantly cell associated. This inverse pattern of HuR and MMP13 staining was restricted to these hypertrophic chondrocytes as MMP13 was also strongly expressed in mineralising bone where cellular HuR was also abundant [Fig. 6(H)].

## Discussion

In this study we begin to examine how RNABPs that are known to modulate post-transcriptional gene expression, affect anabolic and catabolic gene expression in chondrocytes and have identified potentially opposing effects of TTP and HuR.

TTP is a zinc finger-containing protein, which has an established role in the post-transcriptional control of a number of inflammatory cytokines^31^, ^32^, ^33^. We have demonstrated through our knockdown studies that TTP acts as a suppressor of SOX9 expression levels. We have also shown that reduced TTP levels lead to stabilisation of the SOX9 mRNA and that it can bind to AREs within the SOX9 mRNA 3′UTR. This data points to a role for TTP as a modulator of SOX9 mRNA decay. However, the modest scale of the effect that we observe, taken together with our existing understanding of post-transcriptional control of SOX9 indicate that it is likely to contribute to the relative instability of the SOX9 transcript as part of a larger regulatory mechanism. Mice deficient in TTP develop normally but quickly develop a tumor necrosis factor alpha (TNFα)-dependent autoimmune syndrome, which includes the development of inflammatory arthritis as they age^34^, ^35^. The lack of a severe skeletal phenotype in the TTP −/− mice would argue against TTP mediated post-transcriptional regulation having a significant effect on cartilage development; however, it is likely that this form of regulation is subtler and may assist in controlling SOX9 mRNA levels and responsiveness during the maintenance of cartilage homoeostasis. Furthermore, there may be a degree of redundancy in the post-transcriptional process with other factors contributing alongside TTP in regulating SOX9 mRNA. SOX9 forms part of transactivation complexes to regulate gene expression and is known to interact with SOX5 and SOX6 as part of this process. Analysis of the 3′UTR of these genes' human mRNAs indicates that SOX6 in particular has the potential for post-transcriptional regulation that may parallel that of SOX9 (analysis of Genbank accession NM_01145811, data not shown). It contains six UAUUUAU motifs, similar to those that we have shown TTP interacts with in the SOX9 mRNA. Therefore, it would be interesting to develop this work further to clarify how SOX6 is post-transcriptionally controlled into SOX9 in chondrocytes.

In addition to protein binding, interactions of mRNA 3′UTR elements with miRNAs also perform a critical role in post-transcriptional gene control at the level of both mRNA decay and translation regulation. A previous study has indicated that miR-124 can regulate SOX9 mRNA through interactions with sequences in its 3′UTR^36^. This regulation helped to drive the differentiation of progenitor cells inhabiting the subventricular zone stem cell niche. More recent studies have shown that miR-145 is a negative regulator of SOX9 expression in chondrocytes^26^, ^27^. Furthermore, miR-101 can control SOX9 expression levels in hepatocellular carcinoma^37^. The seed sites for each of these miRNAs lie within the SOX9 3′UTR, in the region more proximal to the stop codon than the TTP binding sites. Therefore, it is likely that the major driver of SOX9 mRNA instability is interactions of small RNAs and RNABPs within this region. Our data indicates that TTP contributes to SOX9 mRNA instability, but that it binds to an area of the 3′UTR further downstream of the stop codon and may thus be outside of the main destabilising region. It is possible that it acts a responsive element allowing modulation of SOX9 mRNA decay rates. This would fit well with previous studies demonstrating altered activity of TTP in response to mitogen-activated protein kinase (MAPK) signalling^38^, which is particularly interesting given our previous observations of p38 MAPK meditated regulation of SOX9 by hyperosmolarity^24^.

We chose to examine the expression of catabolic genes in our RNABP knockdown cultures. MMP13 is a collagenase, which is considered to perform a key role in the non-reversible destruction of the type II collagen network in cartilage during osteoarthritis^39^. HuR appears to act as an overall negative regulator of MMP13, as its knockdown leads to elevated MMP13 expression levels. However, MMP13 mRNA is a very stable transcript and HuR levels do not noticeably affect its rate of decay, indicating that this regulation may be indirect. Our further analysis of the influence of HuR on transcriptional repressors of MMP13 provides evidence for a mechanism that could lead to this regulation, as it suppresses the expression of candidates such as RUNX2, SP1 and USF1. It is still not clear whether this is caused by direct modulation of the stability of these mRNAs however, as no affect on RUNX2 and SP1 mRNA stability was noted, whilst in the case of USF1 the affect of lower levels of HuR is to create a less abundant but more stable pool of mRNA. It is likely that HuR's influence over multiple transcripts leads, indirectly to conditions where MMP13 is up or down regulated. HuR was of particular interest to us given that mouse knockout studies have identified a role for it in skeletal development^28^. By examining the distribution of HuR in mouse embryos we have built upon these findings, identifying regional differences in the expression of HuR. It is particularly interesting that despite being a widely expressed protein, we have observed reduced levels in hypertrophic chondrocytes as well as in chondrocytes adjacent to the perichondrium. These regions have been implicated in regulation of long bone growth and mineralisation^40^. Furthermore, HuR in condensing mesenchyme of the future digits at E13.5 demonstrates a “checkerboard” appearance of HuR staining, which could be relevant to the on-going differentiation of this tissue. Further work is now required to identify how the altered levels of HuR we observe contribute to skeletal development.

In conclusion, we have shown that the TTP and HuR can contribute in opposing ways to control the balance of anabolic and catabolic gene expression in human chondrocytes. It is still not clear what the context is for this level of regulation in chondrocyte biology and to what degree these proteins act alongside other factors to control this process. However, post-transcriptional control mechanisms represent a fascinating means of modulation of important chondrocyte regulatory genes, which might provide important targets for the treatment of joint diseases such as osteoarthritis.

## Author contributions

Study design: BM, ST, PC, GBG; Data collection: BM, SE, ST; Data analysis: BM, SE, ST; Preparing manuscript: BM, ST, PC, GBG. ST takes responsibility for the integrity of this work.

## Conflicts of interest

None.

## Figures and Tables

**Table I:** Sequences of primers used for qRT-PCR analysis

Target gene	Forward primer 5′–3′	Reverse primer 5′–3′
GAPDH	ATGGGGAAGGTGAAGGTCG	TAAAAGCAGCCCTGGTGACC
SOX9	TCCAAGCGCATTACCCACTT	GTTGATTTCGCTGCTCCATTTAG
TTP	TCAGCGCTCCCACTCTCG	GGCTCTCGTAGATGGCAGTCAG
HuR	TTCACCACCAGGCGCAGAGA	GAGCCCGCTCATGTGATCGA
KSRP	CCAAGGACTTCAATGACAGAAG	CCTGTTGGATTTTGTTAATTTGTTCA
Aggrecan	TCGAGGACAGCGAGGCC	TCGAGGGTGTAGCGTGTAGAGA
AUF-1	GGTGGTTTTGGTGAGGTGGAATC	CCCACGCCTCTTATTGGTCTTGT
COL2A1	GGCAATAGCAGGTTCACGTACA	CGATAACAGTCTTGCCCCACTT
MMP13	TCCCAGGAATTGGTGATAAAGTAGA	CTGGCATGACGCGAACAATA
RUNX2	GGAGTGGACGAGGCAAGAGTTT	AGCTTCTGTCTGTGCCTTCTGG
USF1	CCCAGGGCTCAGAGGCACTG	GCGTTTCTCATCCCGAGTCGTC
SP1	GGGGCCCAATGGACAGGTCAG	TGAGGCAATGGGTGTGAGAGTGGT
GATA1	TGGAGACTTTGAAGACAGAGCGGCTGAG	GAAGCTTGGGAGAGGAATAGGCTGCTGA

**Table II:** EMSA RNA probe sequences

Probe number	RNA probe sequence∗	Base location in SOX9 mRNA relative to stop codon (base location in full length mRNA)†
1	AUUUUGUUUUUUCUUCUUCUUCUUCUUCCUUAAAGACAUUUAAGCUAAAGGCAACUCGUACCCAAAUUUCCAAGACACA	117–195 (2018–2095)
2	GUAUGUACUGUGUAUGAUUCAUUACCAUUUUGAGGGGAUUUAUACAUAUUUUUAGAUAAAAUUAAAUGCUCUUAUUUUU	451–529 (2352–2429)
3	UCUUACAAAAAGAAAAAAAAAAUCCUGUUGUAUUAACAUUUAAAAACAGAAUUGUGUUAUGUGAUCAGUUUUGGGGGUU	611–689 (2511–2589)
4	UUGGGGGUUAACUUUGCUUAAUUCCUCAGGCUUUGCGAUUUAAGGAGGAGCUGCCUUAAAAAAAAAUAAAGGCCUUAUU	681–759 (2581–2659)
5	AGCAGUUAACCUUCAAGACAUUCCACUUGCUAAAAUUAUUUAUUUUGUAAGGAGAGGUUUUAAUUAAAACAAAAAAAAA	1088–1166 (2988–3066)
6	GUUAAAUUAUGUUCUUAACUGUAACCAGUUUUUUUUUAUUUAUCUCUUUAAUCUUUUUUUAUUAUUAAAAGCAAGUUUC	1248–1326 (3149–3226)
7	GAUUGCUUUUUAAAAAAGACAGCAAACUUUUUUUUUUAUUUAAAAAAAGAUAUAUUAACAGUUUUAGAAGUCAGUAGAA	1675–1753 (3576–3653)
8	UUUUAAAAAGAUACUUCUGUAACUUAAGAAACCUGGCAUUUAAAUCAUAUUUUGUCUUUAGGUAAAAGCUUUGGUUUGU	1812–1890 (3712–3790)

∗ AUUUA pentamer sequences in each probe are underlined.

† Reference sequence used Entrez accession number NM_000346.

## References

[1] Hardingham T., Tew S., Murdoch A. (2002). Tissue engineering: chondrocytes and cartilage. Arthritis Res.

[2] Akiyama H. (2008). Control of chondrogenesis by the transcription factor Sox9. Mod Rheumatol.

[3] Billinghurst R.C., Dahlberg L., Ionescu M., Reiner A., Bourne R., Rorabeck C. (1997). Enhanced cleavage of type II collagen by collagenases in osteoarthritic articular cartilage. J Clin Invest.

[4] Karsdal M.A., Madsen S.H., Christiansen C., Henriksen K., Fosang A.J., Sondergaard B.C. (2008). Cartilage degradation is fully reversible in the presence of aggrecanase but not matrix metalloproteinase activity. Arthritis Res Ther.

[5] Tew S.R., Clegg P.D., Brew C.J., Redmond C.M., Hardingham T.E. (2007). SOX9 transduction of a human chondrocytic cell line identifies novel genes regulated in primary human chondrocytes and in osteoarthritis. Arthritis Res Ther.

[6] Gruber H.E., Norton H.J., Ingram J.A., Hanley E.N. (2005). The SOX9 transcription factor in the human disc: decreased immunolocalization with age and disc degeneration. Spine (Phila Pa 1976).

[7] Tew S.R., Li Y., Pothacharoen P., Tweats L.M., Hawkins R.E., Hardingham T.E. (2005). Retroviral transduction with SOX9 enhances re-expression of the chondrocyte phenotype in passaged osteoarthritic human articular chondrocytes. Osteoarthritis Cartilage.

[8] Paul R., Haydon R.C., Cheng H., Ishikawa A., Nenadovich N., Jiang W. (2003). Potential use of Sox9 gene therapy for intervertebral degenerative disc disease. Spine.

[9] Johnson A.R., Pavlovsky A.G., Ortwine D.F., Prior F., Man C.F., Bornemeier D.A. (2007). Discovery and characterization of a novel inhibitor of matrix metalloprotease-13 that reduces cartilage damage in vivo without joint fibroplasia side effects. J Biol Chem.

[10] Bagheri-Fam S., Barrionuevo F., Dohrmann U., Gunther T., Schule R., Kemler R. (2006). Long-range upstream and downstream enhancers control distinct subsets of the complex spatiotemporal Sox9 expression pattern. Dev Biol.

[11] Bagheri-Fam S., Ferraz C., Demaille J., Scherer G., Pfeifer D. (2001). Comparative genomics of the SOX9 region in human and Fugu rubripes: conservation of short regulatory sequence elements within large intergenic regions. Genomics.

[12] Colter D.C., Piera-Velazquez S., Hawkins D.F., Whitecavage M.K., Jimenez S.A., Stokes D.G. (2005). Regulation of the human Sox9 promoter by the CCAAT-binding factor. Matrix Biol.

[13] Piera-Velazquez S., Hawkins D.F., Whitecavage M.K., Colter D.C., Stokes D.G., Jimenez S.A. (2007). Regulation of the human SOX9 promoter by Sp1 and CREB. Exp Cell Res.

[14] Ushita M., Saito T., Ikeda T., Yano F., Higashikawa A., Ogata N. (2009). Transcriptional induction of SOX9 by NF-kappaB family member RelA in chondrogenic cells. Osteoarthritis Cartilage.

[15] Samuel S., Beifuss K.K., Bernstein L.R. (2007). YB-1 binds to the MMP-13 promoter sequence and represses MMP-13 transactivation via the AP-1 site. Biochim Biophys Acta.

[16] Hashimoto K., Otero M., Imagawa K., de Andres M.C., Coico J.M., Roach H.I. (2013). Regulated transcription of human matrix metalloproteinase 13 (MMP13) and interleukin-1beta (IL1B) genes in chondrocytes depends on methylation of specific proximal promoter CpG sites. J Biol Chem.

[17] Huang W., Zhou X., Lefebvre V., de Crombrugghe B. (2000). Phosphorylation of SOX9 by cyclic AMP-dependent protein kinase A enhances SOX9's ability to transactivate a Col2a1 chondrocyte-specific enhancer. Mol Cell Biol.

[18] Hattori T., Eberspaecher H., Lu J., Zhang R., Nishida T., Kahyo T. (2006). Interactions between PIAS proteins and SOX9 result in an increase in the cellular concentrations of SOX9. J Biol Chem.

[19] Van Wart H.E., Birkedal-Hansen H. (1990). The cysteine switch: a principle of regulation of metalloproteinase activity with potential applicability to the entire matrix metalloproteinase gene family. Proc Natl Acad Sci U S A.

[20] Cawston T.E., Galloway W.A., Mercer E., Murphy G., Reynolds J.J. (1981). Purification of rabbit bone inhibitor of collagenase. Biochem J.

[21] Barreau C., Paillard L., Osborne H.B. (2005). AU-rich elements and associated factors: are there unifying principles?. Nucleic Acids Res.

[22] Guhaniyogi J., Brewer G. (2001). Regulation of mRNA stability in mammalian cells. Gene.

[23] Tew S.R., Hardingham T.E. (2006). Regulation of SOX9 mRNA in human articular chondrocytes involving p38 MAPK activation and mRNA stabilization. J Biol Chem.

[24] Tew S.R., Peffers M.J., McKay T.R., Lowe E.T., Khan W.S., Hardingham T.E. (2009). Hyperosmolarity regulates SOX9 mRNA posttranscriptionally in human articular chondrocytes. Am J Physiol Cell Physiol.

[25] Tew S.R., Clegg P.D. (2011). Analysis of post transcriptional regulation of SOX9 mRNA during in vitro chondrogenesis. Tissue Eng Part A.

[26] Martinez-Sanchez A., Dudek K.A., Murphy C.L. (2012). Regulation of human chondrocyte function through direct inhibition of cartilage master regulator SOX9 by microRNA-145 (miRNA-145). J Biol Chem.

[27] Yang B., Guo H., Zhang Y., Chen L., Ying D., Dong S. (2011). MicroRNA-145 regulates chondrogenic differentiation of mesenchymal stem cells by targeting Sox9. PLoS One.

[28] Katsanou V., Milatos S., Yiakouvaki A., Sgantzis N., Kotsoni A., Alexiou M. (2009). The RNA-binding protein Elavl1/HuR is essential for placental branching morphogenesis and embryonic development. Mol Cell Biol.

[29] Livak K.J., Schmittgen T.D. (2001). Analysis of relative gene expression data using real-time quantitative PCR and the 2(−Delta Delta C(T)) method. Methods.

[30] Worthington M.T., Pelo J.W., Sachedina M.A., Applegate J.L., Arseneau K.O., Pizarro T.T. (2002). RNA binding properties of the AU-rich element-binding recombinant Nup475/TIS11/tristetraprolin protein. J Biol Chem.

[31] Van Tubergen E., Vander Broek R., Lee J., Wolf G., Carey T., Bradford C. (2011). Tristetraprolin regulates interleukin-6, which is correlated with tumor progression in patients with head and neck squamous cell carcinoma. Cancer.

[32] Tudor C., Marchese F.P., Hitti E., Aubareda A., Rawlinson L., Gaestel M. (2009). The p38 MAPK pathway inhibits tristetraprolin-directed decay of interleukin-10 and pro-inflammatory mediator mRNAs in murine macrophages. FEBS Lett.

[33] Carballo E., Lai W.S., Blackshear P.J. (1998). Feedback inhibition of macrophage tumor necrosis factor-alpha production by tristetraprolin. Science.

[34] Taylor G.A., Carballo E., Lee D.M., Lai W.S., Thompson M.J., Patel D.D. (1996). A pathogenetic role for TNF alpha in the syndrome of cachexia, arthritis, and autoimmunity resulting from tristetraprolin (TTP) deficiency. Immunity.

[35] Carballo E., Blackshear P.J. (2001). Roles of tumor necrosis factor-alpha receptor subtypes in the pathogenesis of the tristetraprolin-deficiency syndrome. Blood.

[36] Cheng L.C., Pastrana E., Tavazoie M., Doetsch F. (2009). miR-124 regulates adult neurogenesis in the subventricular zone stem cell niche. Nat Neurosci.

[37] Zhang Y., Guo X., Xiong L., Kong X., Xu Y., Liu C. (2012). MicroRNA-101 suppresses SOX9-dependent tumorigenicity and promotes favorable prognosis of human hepatocellular carcinoma. FEBS Lett.

[38] Mahtani K.R., Brook M., Dean J.L., Sully G., Saklatvala J., Clark A.R. (2001). Mitogen-activated protein kinase p38 controls the expression and posttranslational modification of tristetraprolin, a regulator of tumor necrosis factor alpha mRNA stability. Mol Cell Biol.

[39] Little C.B., Barai A., Burkhardt D., Smith S.M., Fosang A.J., Werb Z. (2009). Matrix metalloproteinase 13-deficient mice are resistant to osteoarthritic cartilage erosion but not chondrocyte hypertrophy or osteophyte development. Arthritis Rheum.

[40] Vortkamp A., Lee K., Lanske B., Segre G.V., Kronenberg H.M., Tabin C.J. (1996). Regulation of rate of cartilage differentiation by Indian hedgehog and PTH-related protein. Science.

